# Editor’s Highlight: Subvisible Aggregates of Immunogenic Proteins Promote a Th1-Type Response

**DOI:** 10.1093/toxsci/kfw121

**Published:** 2016-06-30

**Authors:** Kirsty D. Ratanji, Rebecca J. Dearman, Ian Kimber, Robin Thorpe, Meenu Wadhwa, Jeremy P. Derrick

**Affiliations:** *Faculty of Life Sciences, The University of Manchester, C1256, Michael Smith Building, Dover St, Manchester M13 9PT, UK;; ^†^National Institute for Biological Standards and Control, Potters Bar, Hertfordshire EN6 3QG

**Keywords:** immunogenicity, biotherapeutic, protein aggregation, scFv, OVA.

## Abstract

Protein aggregation is associated with enhanced immunogenicity of biotherapeutics. As a result, regulatory guidelines recommend screening for aggregation during bioprocessing. However, the mechanisms underlying the enhanced immunogenicity of aggregates are poorly understood. In the investigations described herein, the immunogenicity in mice of a humanized single chain variable antibody fragment (scFv) purified after expression in *Escherichia coli* has been examined. Reproducible scFv aggregates were obtained within the subvisible particle size range (mean diameter 2 µm) using thermal and mechanical stresses. Intraperitoneal immunization of BALB/c strain mice with 1 mg/ml of aggregated or monomeric scFv induced similar IgG and IgG1 antibody responses. In contrast, aggregate preparations stimulated significantly higher levels of anti-scFv IgG2a antibody than did the monomer. In comparative studies, aggregates of ovalbumin (OVA) within the subvisible particle size range were prepared by stir stress, and their immunogenicity compared with that of monomeric OVA in mice. Aggregated and monomeric OVA induced similar anti-OVA IgG and IgG1 antibody responses, whereas IgG2a antibody levels were significantly higher in aggregate-immunized mice. Furthermore, cytokine profiles in supernatants taken from splenocyte-dendritic cell co-cultures were consistent with aggregated preparations inducing a T helper (Th) 1-type response. Aggregated proteins within the subvisible range were therefore shown to induce a preferential Th1 type response, whereas monomeric proteins elicited a selective Th2 response. These data indicate that protein aggregation can impact on both the vigor and quality of immune responses.

Unwanted immunogenicity of recombinant biotherapeutics has the potential to impact upon drug efficacy and patient safety. Even biotherapeutics consisting of completely human-derived sequences have the potential to elicit an immunogenic response in patients. For example, immunogenicity of Interferon-β (Ross *et al.*, 2000) and Adalimumab (Bartelds *et al.*, 2011) have resulted in compromised drug efficacy in the clinic. Eprex (epoetin α) and thrombopoietin are examples of biotherapeutics that have resulted in adverse events in some patients, due to the development of neutralizing antibodies (Casadevall *et al.*, 2002; Li *et al.*, 2001). Eprex is a well-documented example of immunogenicity and, while the causative factor has not been identified, various factors including micelle formation, and change of formulation, have been implicated (Schellekens and Jiskoot, 2006).

Various treatment, patient, and product-related factors may contribute to biotherapeutic immunogenicity (Singh, 2011). Protein aggregation is a product-related factor that has the potential to enhance immunogenicity (Ratanji *et al.*, 2014), and regulatory authorities therefore recommend screening for and minimizing the presence of aggregates in parenteral biotherapeutic products (European Directorate for the Quality of Medicine [EDQM]. 2010; Food and Drug Administration [FDA], 2014).

Proteins are subject to many stresses at different stages in bioprocessing and manufacture, from protein expression in the host cell system to storage and handling. Physical and chemical stressors such as mechanical stress, pH, elevated temperature, high-protein concentrations, and repeated freeze-thaw can all result in partial unfolding and aggregation (Frokjaer and Otzen, 2005). Since the immunogenicity of proteins is confounded by a number of potential variables, including route of administration, formulation, product container and host cell protein impurities, it can be challenging to determine the influence of aggregation *per se* on protein immunogenicity. Furthermore, protein aggregates can be diverse in their biophysical and biochemical characteristics. Aggregate size is one variable that could influence immunogenicity. Aggregates are known to range in size from oligomers of nanometer dimensions, to subvisible and visible particulates (Narhi *et al.*, 2012), but it is not clear how such variations in size affect the immune response.

In recent years research into protein aggregate immunogenicity has increased. *In vitro* approaches using human peripheral blood mononuclear cells (Joubert *et al.*, 2012; Telikepalli *et al.*, 2015) or dendritic cells (DCs) (Rombach-Riegraf *et al.*, 2014) have had some success when used to screen aggregates for their potential to influence cytokine expression profiles and surface marker expression. However, although *in vitro* assays may be useful in screening for immunogenicity to select lead candidates, they do not provide a holistic appreciation of the immunogenicity of a product, nor do they necessarily facilitate characterization of the relevant immunological mechanisms. In addition, *in vivo* approaches have been adopted to compare and contrast immune responses elicited by aggregated proteins and their monomeric counterparts. A common approach is to measure antibody responses provoked in mice by immunizations with the monomeric or aggregated protein (Freitag *et al.*, 2015; Shomali *et al.*, 2014). Furthermore, transgenic therapeutic-tolerant mouse models have been used to assess the ability of aggregates to break immunological tolerance (Bessa *et al.*, 2015; Braun *et al.*, 1997; Kijanka *et al.*, 2015).

However, to our knowledge, previous investigations have not sought to evaluate the quality as well as the vigor of induced immune responses *in vivo*, using both antibody isotyping and *ex vivo* cellular assays. Functional subpopulations of T helper (Th) cells provide a mechanism for the development of different qualities of immune responses, depending on the nature of the immune challenge. Th1 cells are responsible for cell-mediated immunity, whereas Th2 cells produce cytokines that promote humoral responses (Abbas *et al.*, 1996). A more comprehensive analysis of the quality of induced immune responses (ie, Th1 or Th2) could provide a better understanding of the fundamental mechanisms underpinning aggregate immunogenicity.

The aim of the investigations described here was to characterize the relationships between aspects of protein aggregation, including aggregate size, with the vigor and quality of induced immune responses in mice. The aim was not to develop a method for predicting the potential of a protein to provoke an immune response in humans, but rather to investigate the influence of aggregation on immune responses. For this purpose a humanized single chain variable antibody fragment (scFv) and ovalbumin (OVA) were selected as test proteins. In using proteins that are foreign to mice, a baseline level of immunogenicity was expected to be achieved with monomers, and changes to this baseline with aggregation were studied. For both proteins, preparations of protein aggregates within distinct size ranges were produced using thermal and mechanical stress and the immunogenicity profile assessed in BALB/c strain mice in terms of antibody production and cytokine expression, in comparison with their monomeric counterparts.

## MATERIALS AND METHODS

### scFv Purification

A humanized scFv, as reported by (Edwardraja *et al.*, 2010), was cloned into a pET-22b vector in Shuffle T7 express *Escherichia coli* cells (New England Biolabs, Beverly, Massachusetts). Transformants were cultured at 30 °C to an optical density (OD) of 0.8 at 600 nm, induced with isopropyl β-D-1-thiogalactopyranoside and incubated overnight at 16 °C. Cell pellets were resuspended, sonicated and centrifuged at 28 672 g for 30 min. scFv was purified from supernatants using DEAE (diethylaminoethanol) Sepharose anion exchange chromatography, followed by Protein A affinity and size exclusion chromatography.

### Generation of Aggregates

#### scFv

Purified monomeric scFv was diluted to 1 mg/ml in Dulbeccos phosphate buffered saline (PBS) without Ca ^+^ ^2^ or Mg ^+^ ^2^ (Sigma-Aldrich, St Louis, Missouri) and stressed by heating at 40 °C for 25 min. To induce stir-stressed aggregates 1 ml of 1 mg/ml purified scFv was stirred with an 8 × 2 mm Teflon stirrer bar in a 5 ml glass tube for 6 h. *OVA:* Lyophilized OVA (Sigma-Aldrich) was diluted to 1 mg/ml in Dulbeccos PBS and stirred in a volume of 1ml in a 5 ml glass tube for 24–28 h. Dynamic light scattering (DLS) was used to monitor aggregation status and stirring stopped once the desired subvisible size range achieved.

### Endotoxin Measurement

The endotoxin content of protein preparations was measured chromatographically by limulus amebocyte assay according to the manufacturer's instructions (Cambrex BioSciences, Wokingham, UK). Endotoxin levels were <100 EU/mg of protein; levels which have been shown previously to be without impact on *in vivo* antibody responses (Dearman and Kimber, 2007).

### Analysis of Aggregates

Measurements of DLS were performed with a Malvern Zetasizer Nano ZS ZEN3600 (Malvern, Herrenberg, Germany) equipped with a 633 nm laser. Each sample (70 µl) was measured in a Suprasil quartz cuvette (Hellma GmbH, Muellheim, Germany) with a path length of 3 mm and 200–2500 nm spectral range. Monomeric and stressed samples at 1 mg/ml were measured at 25 °C to determine the volume-based average protein particle diameter in solution.

### Animal Experiments

Female BALB/c strain mice were used for these experiments (Envigo, Bicester, UK). All procedures were carried out in accordance with the Animals (Scientific Procedures) Act 1986, and approved by Home Office licence. Mice were immunized by intraperitoneal (ip) injection (or subcutaneous [sc] injection) with 250 µl of 1 or 0.1 mg/ml protein (monomeric or aggregate) in PBS on days 0 and 7 and exsanguinated on day 14. In some experiments mice received an additional immunization on day 14 and were terminated on day 21. Spleens and serum were isolated for evaluation.

### Generation and Culture of Murine Bone Marrow Derived DC

Murine bone marrow (BM) derived DC (BMDC) were generated using a previously described method (Lutz *et al.*, 1999). Briefly, BM was extracted by flushing the femurs and tibias with PBS. The cell suspension was centrifuged at 112 g for 5 min. The pellet was then resuspended in warmed culture medium (RPMI 1640, GIBCO, Paisley, UK), supplemented with 10% fetal calf serum (FCS) (PAA laboratories, GmbH, Austria) containing 400 µg/ml penicillin/streptomycin, 292 µg/ml L-glutamine, 0.05 mM 2-mercaptoethanol and 20 ng/ml granulocyte macrophage-colony stimulating factor (Miltenyi Biotech, Bisley, UK). Viable cell counts were performed by trypan blue exclusion (0.5%, Sigma-Aldrich). Cells were cultured at approximately 2 × 10^6^ cells per 100 × 15mm petri dish at 37 °C in a humidified atmosphere of 5% CO_2_ in air_._ Medium was refreshed every 3 days, and cells harvested by gentle agitation. BMDC were used in co-culture with splenocytes on day 7.

### Splenocyte-BMDC Co-Culture

Single cell suspensions of splenocytes were prepared by mechanical disaggregation. Red blood cells were lysed by incubation in 0.85% ammonium chloride for 3 min. Splenocytes were washed and resuspended in culture medium, supplemented with 10% FCS containing 400 µg/ml penicillin/streptomycin, 292 µg/ml L-glutamine and cultured at 3 × 10^5^ cells per well in 96 well round bottomed tissue culture plates, alone or in co-culture with 3 × 10^4^ BMDC per well. Triplicate wells per individual mouse were cultured with monomeric or aggregated protein at 100 μg/ml, or with the T cell mitogen Concanavalin A (conA; Sigma-Aldrich) as a positive control at 2 μg/ml, or with an equal volume of medium alone for negative control wells and cultured at 37^o^C in a humidified atmosphere of 5% CO_2_ for 72–144 h. Supernatants were harvested by centrifugation.

#### 3H-Thymidine incorporation assay

In parallel, aliquots of cells were pulsed with 0.2 MBq ^3^H- thymidine (^3^HTdR) (PerkinElmer, Waltham, Massachusetts, USA) per well 24 h before harvesting. Cells were harvested onto glass fibre filter mats with a multichannel semi-automated harvesting device (Titertek, Skatron AS, Lierbyen, Norway). Incorporation of ^3^HTdR was measured as disintegrations per minute (DPM) in a liquid scintillation cocktail (Fisher Scientific, Loughborough, UK). Standard error of mean (SEM) was calculated from averages of 3 replicate wells.

#### Cytokine enzyme-linked immunosorbent assay: interferon-γ, interleukin-13, and interleukin-4

Splenocyte culture supernatants were tested for Interferon-γ (IFNγ), Interleukin-13 (IL-13) and Interleukin-4 (IL-4) protein using specific enzyme-linked immunosorbent assay (ELISA) Duosets from R&D Systems (R&D Systems, Minneapolis, USA). The lower limits of accurate detection were: 31.25 pg/ml for IFNγ, 62.5 pg/ml for IL-13 and 15.6 pg/ml for IL-4. ELISAs were performed following the manufacturer's instructions.

### ELISA for protein-Specific Antibody Classes and Subclasses

Plastic Maxisorb plates (Nunc, Copenhagen, Denmark) were coated with 10 µg/ml of protein in PBS overnight at 4 °C. Plates were blocked with 2% bovine serum albumin (BSA)/PBS (Sigma Aldrich) at 37 °C for 30 min. Doubling dilutions of serum samples were added (starting dilution 1 in 32 or 1in 64 for anti-IgG; 1 in 128 or 1in 64 for anti IgM antibody analyses) in 1% BSA/PBS (as a negative control naïve mouse serum [NMS] samples were added to plates) and incubated for 3 h at 4 °C. Plates were incubated for 2 h at 4 °C with horseradish peroxidise labeled sheep anti-mouse IgG diluted 1 in 4000, sheep anti-mouse IgG1 diluted 1: 2000, sheep anti-mouse IgG2a diluted 1: 1000 (all Serotec, Oxfordshire) or goat anti-mouse IgM diluted 1: 6000 (Invitrogen, Paisley, UK). Plates were washed between incubations with 0.05% Tween 20 in PBS. Plates were incubated with substrate *o*-phenylenediamine and urea hydrogen peroxide for 15 min and reactions were stopped with 0.5 M citric acid. Absorbance was read at 450 nm using an automated reader (Multiscan, Flow Laboratories, Irvine, Ayrshire, UK). Data are displayed as OD450 nm values and mean antibody titers. Titer was calculated as the maximum dilution of serum at which an OD450 reading of 0.3 or above is recorded (3 times reagent blank [all reagents except for serum] reading of 0.1). If an OD 450 nm reading of 3 times the reagent blank at the highest serum concentration was not achieved, a nominal titer value of 16 was assigned.

### Statistical Analyses

Statistical analyses were performed using the software Graphpad Prism 6. Analysis of variance (ANOVA) was used to determine statistical significance of differences between groups. Experiments were analyzed by non-parametric 1- or 2-way ANOVA followed by the Tukey *post hoc* test (*P < .05, **P < .01, ***P < .001, ****P < .0001).

## RESULTS

### Monomeric and Heat Stressed Aggregates of scFv Induce Differential Antibody Responses

**FIG. 1:**
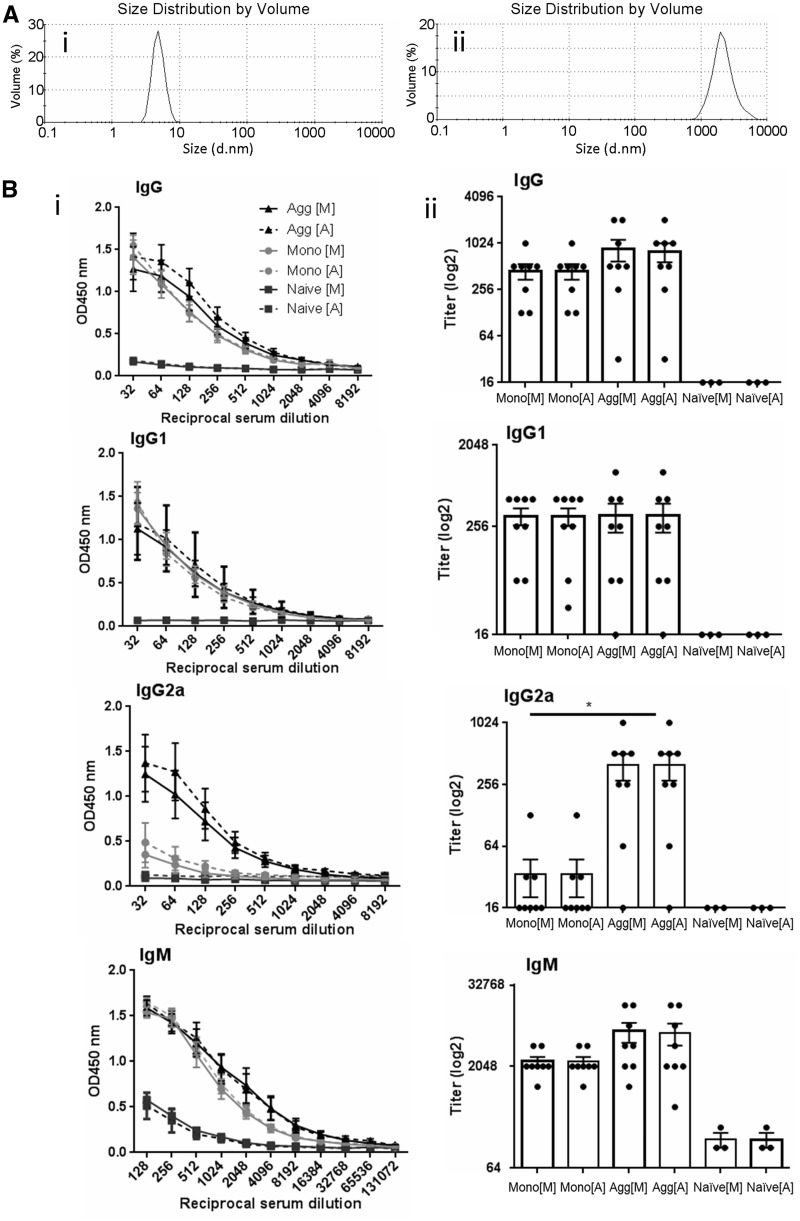
Characterization of immune responses to scFv: comparisons of monomer and heat stressed aggregates. scFv at 1 mg/ml in PBS pH 7 was subjected to heat treatment for 25 min at 40 °C. **A**, The mean particle diameter was measured by DLS before (i) and after (ii) the 40 °C incubation. **B,** Mice were immunized by ip injection with 250 µl of 1 mg/ml monomer or heat aggregated scFv on days 0 and 7 and serum isolated on day 14 (2 independent experiments; n = 5 and n = 3 per group). Doubling dilutions of serum samples from scFv monomer (Mono) and aggregate (Agg) immunized animals and negative control naïve serum samples were analyzed against both scFv substrate proteins (vs monomer [M] and vs aggregated protein [A]) by ELISA for IgG, IgG1, IgG2a, and IgM anti-scFv antibody content. (i) Data are displayed as OD450 nm (±SEM) for each reciprocal serum dilution (32–8192 for IgG antibodies; 128–13 1072 for IgM antibodies) (ii) Data are displayed with respect to antibody titer (log2) calculated as the lowest serum dilution at which 3x the ELISA substrate blank OD450 nm reading was reached. Individual titers are displayed with overall mean and SEM. Statistical significance of differences in antibody detection between all sera groups against each substrate was calculated using a 1-way ANOVA (*P < .05).

To produce aggregates by heat stress, 1 mg/ml scFv in PBS was incubated at 40 °C for 25 min and DLS was employed to observe changes in mean particle diameter. A reproducible increase in size from 7 nm to 1000–3000 nm was observed (Fig. 1A). Aggregate and monomer preparations were also shown to be stable by DLS following 2 freeze/thaw cycles. Initial experiments with the scFv monomer demonstrated that a concentration of 0.1 mg/ml resulted in a weak or undetectable IgG antibody response in some animals. A robust, detectable response was required in these experiments for comparison to the aggregated protein (data not shown). A 1 mg/ml dose was found to provide a consistent antibody response, so for all further experiments this concentration was used.

Preparations of monomer and aggregate protein (1 mg/ml) were administered via ip injection to mice on days 0 and 7 (2 independent experiments; n = 5 and n = 3 per group) and sera isolated on day 14. To compare immune responses induced by monomer and aggregate, the presence of anti-scFv IgG, IgG1, IgG2a, and IgM antibody in serum samples was analyzed by ELISA (Fig. 1B).

Analysis of the serum dilution curves and antibody titers revealed that there were no false positive IgG, IgG1 or IgG2a antibody readouts for scFv in the negative control NMS samples, regardless of whether aggregated or monomeric protein was used as the substrate (Fig. 1B). Although comparatively low levels of IgM antibody were apparently present in naïve sera, this was due to non-specific binding that was also observed with BSA and OVA substrates (data not shown). Relatively high level expression levels of anti-scFv IgG, IgG1, and IgM antibodies were found in sera isolated from monomer or aggregate immunized mice, with virtually identical titration curves irrespective of whether immunization was with the monomer or the aggregated form, or whether the substrate was monomeric or aggregated. In contrast, only immunization with the aggregated form of scFv resulted in a high level of IgG2a antibody production. Further investigations revealed that this was a robust and reproducible finding, with this same pattern observed in each of the 2 independent experiments. Thus, in each experiment, equivalent anti-scFv IgG, IgG1, and IgM antibody titers were recorded following immunization with monomer or aggregated protein, and identical titers were observed regardless of which material was used as substrate in the ELISA. However, a significantly higher titer IgG2a antibody response was recorded in sera from aggregate compared with monomer immunized mice (*P < .05).

### Aggregation and IgG2a Skewing Is Independent of Either Dose or Method of Aggregation

**FIG. 2:**
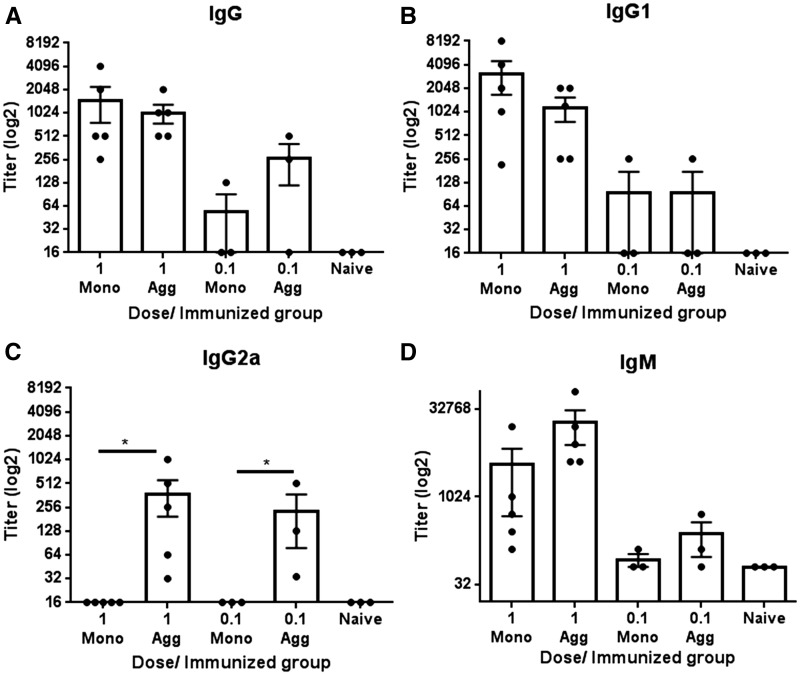
Antibody response to scFv: influence of dose and aggregation status. Mice were immunized by ip injection with 250 µl of 1 (n = 5) or 0.1 mg/ml (n = 3) monomer or heat aggregated scFv on days 0, 7 and 14 and serum isolated on day 21. Doubling dilutions of serum samples (starting dilution 1 in 32) from scFv monomer (Mono) and aggregate (Agg) immunized animals and negative control NMS samples were analyzed against scFv substrate proteins (versus immunizing protein only results are shown) by ELISA for IgG **(A)**, IgG1 **(B),** IgG2a **(C)**, and IgM **(D)** anti-scFv antibody content. Data are displayed with respect to antibody titer (log2), calculated as the lowest serum dilution at which 3× the ELISA substrate blank OD450nm reading was reached. Individual titers are displayed with overall mean and SEM. Statistical significance of differences in antibody binding between all treatment groups against substrate were calculated using a 1-way ANOVA (*P < .05).

In subsequent experiments, the ability of aggregated scFv to induce IgG2a antibody skewing was confirmed. Mice (n = 3-5) were immunized with monomer or heat stressed aggregate at 1 or 0.1 mg/ml using a more vigorous dosing regimen with an additional immunization (day 14), and termination on day 21 (Fig. 2).

For ELISA analyses of serum samples, the substrate used was the same as the immunizing material, as it had already been confirmed that identical responses were recorded with monomeric and aggregated scFv substrates (Fig. 1B). Sera isolated from mice immunized 3 times with monomer or aggregate displayed comparable responses to those observed in mice immunized twice (Fig. 1). Thus, comparable IgG and IgG1 antibody responses were recorded for both forms of protein, but the aggregate protein provoked significantly (*P < .05) higher titer IgG2a antibody responses. Although IgM antibody titers were not significantly different, the maximal OD values recorded in the ELISA were significantly (**P < .01) different between monomer and aggregate immunized mice. Furthermore, mice immunized with a considerably lower dose (0.1 mg/ml) of protein displayed a similar trend, although overall titers were lower compared with the higher dose. In addition, a comparison of ip and sc routes of administration was conducted. Results from studies using these different routes of administration were comparable (Supplementary Fig. S1): only the aggregated material stimulated robust IgG2a antibody responses, regardless of the route of exposure.

**FIG. 3:**
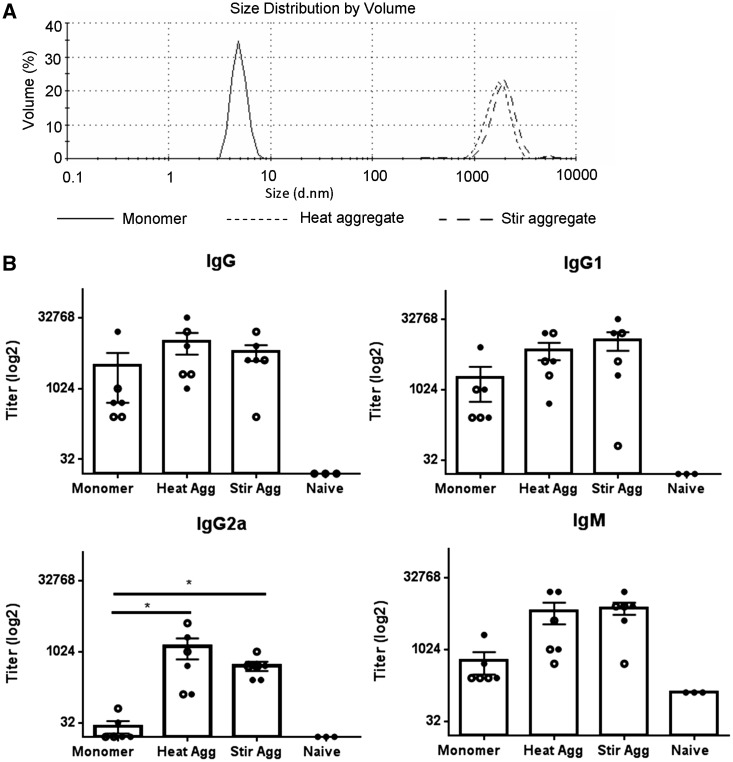
Antibody responses to scFv monomer, heat stressed and stir stressed aggregates. ScFv at 1 mg/ml in PBS pH 7 was subjected to heat treatment for 25 min at 40 °C for thermal stress. To induce stir stress samples were stirred for 6 h at room temperature. **A,** The mean particle diameter was measured by DLS using the Malvern zetasizer before and after the 40 °C incubation or stir stress. **B,** Mice (n = 6) were immunized by ip injection with 250 µl of 1 mg/ml monomer or heat (Heat Agg) or stir aggregated (Stir Agg) scFv on days 0, 7, and 14 and serum isolated on day 21. Doubling dilutions of serum samples (starting dilution 1 in 32 for IgG and 1 in 128 for IgM) from immunized animals and negative control NMS samples were analyzed against scFv substrate proteins (vs immunizing protein only results are shown) by ELISA for IgG, IgG1, IgG2a, and IgM anti-scFv antibody content. Data are displayed with respect to antibody titer (log2), calculated as the lowest serum dilution at which 3× the ELISA substrate blank OD450 nm reading was reached. Individual titers (open and closed symbols) are displayed for 2 independent experiments (n = 3 per group), with overall mean and SEM. Statistical significance of differences in antibody detection between all treatment groups against substrate were calculated using a 1-way ANOVA (*P < .05).

The aggregates studied thus far were formed using thermal stress, and were found to consistently induce higher titer IgG2a antibody responses than did the monomeric protein. Stir stress was employed to determine whether scFv aggregates within a similar size range could be formed using a mechanical stress method. The mean protein particle diameter of aggregates was analyzed by DLS (Fig. 3A), from which it was apparent that aggregates produced using stir stress were similar to heat stressed aggregates. Stir stressed aggregates also had a mean particle diameter of 1000–3000 nm and were stable following 2 freeze/thaw cycles. Anti-scFv antibody production patterns were identical, irrespective of whether stir aggregate or monomer was used as a substrate in the ELISA (data not shown). Monomer and aggregate protein preparations formed using heat or stir stress were administered via ip injection to mice on day 0, 7 and 14 (2 independent experiments; n = 3 in each) and sera isolated on day 21.

Sera isolated from monomer, heat and stir stressed aggregate immunized mice induce comparable IgG and IgG1 antibody titers (Fig. 3). In contrast, IgG2a titers were significantly (*P < .05) higher in animals exposed to heat or stir stressed protein compared with monomer immunized mice. IgM antibody titers were somewhat higher in aggregate compared with monomer immunized mice and, although titers were not significantly different, maximal OD values recorded at the top serum dilution were significantly different (***P < .001) between monomer and both groups of aggregate immunized mice.

### Aggregation of the scFv Induced Differential Cellular Responses: Proliferation and Cytokine Production

**FIG. 4:**
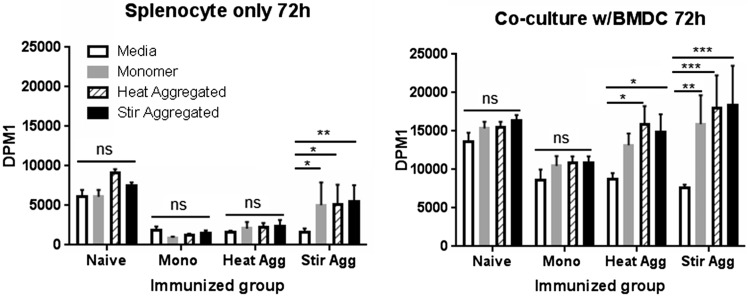
Splenocyte culture ^3^H-thymidine incorporation following *ex vivo* scFv challenge. Splenocytes from monomer (Mono), heat (Heat Agg) or stir aggregated (Stir Agg) scFv immunized (as described in Figure 3 legend), or naïve* mice (n = 3 per group) were cultured alone and in co-culture with BMDC and challenged with 100 µg/ml monomeric scFv, heat or stir aggregated scFv or with media alone. Cells were pulsed with ^3^H-thymidine 24 h before harvesting at 72 h and β scintillation counting. Each culture condition was performed in triplicate for splenocytes derived from each individual animal and a mean calculated. Group mean proliferation measured as DPM is shown ±SEM. Statistical significance of differences were calculated using a 2-way ANOVA (*P < .05, ** *P* < .01, *** *P* < .001). *Experiments with naïve mice were conducted independently but utilized the same batches of mice and protein.

As antibody production profiles suggested that aggregated protein was inducing Th1 type skewing (associated with selective IgG2a antibody responses) we sought to examine this at the cellular level. To this end, antigen-driven proliferation and cytokine production by cultured lymphocytes prepared from the spleens of immunized mice were measured. Splenocytes from immunized and naïve mice were cultured either alone or in the presence of BMDC, the latter included to enhance assay sensitivity. Cultures were primed with monomeric or aggregated scFv and proliferation measured using ^3^H-thymidine incorporation at a 72 h time point (Fig. 4).

Splenocyte only cultures derived from naïve and monomer or heat aggregate immunized mice did not proliferate significantly more in response to stimulation with scFv in any of the formats compared with medium alone. Only stir aggregate immunized mouse splenocytes displayed any activity by responding more effectively to stimulation with all of the scFv preparations (Fig. 4). Interestingly, the nature of the scFv used for antigenic stimulation in culture did not impact on proliferative activity. It should be noted that the baseline level of proliferation for naïve splenocytes was somewhat higher than those observed for cells from the immunized mice; due to logistics the naive cell cultures were not conducted concurrently. The addition of BMDC in co-culture with the splenocytes resulted in improved assay sensitivity, such that both heat and stir aggregate immunized mouse cell cultures responded significantly to scFv compared with medium alone. Again, the nature of the scFv used did not impact on proliferative activity. Naïve and monomer immunized mouse splenocyte cultures did not respond to scFv in culture, regardless of the presence of BMDC.

**FIG. 5:**
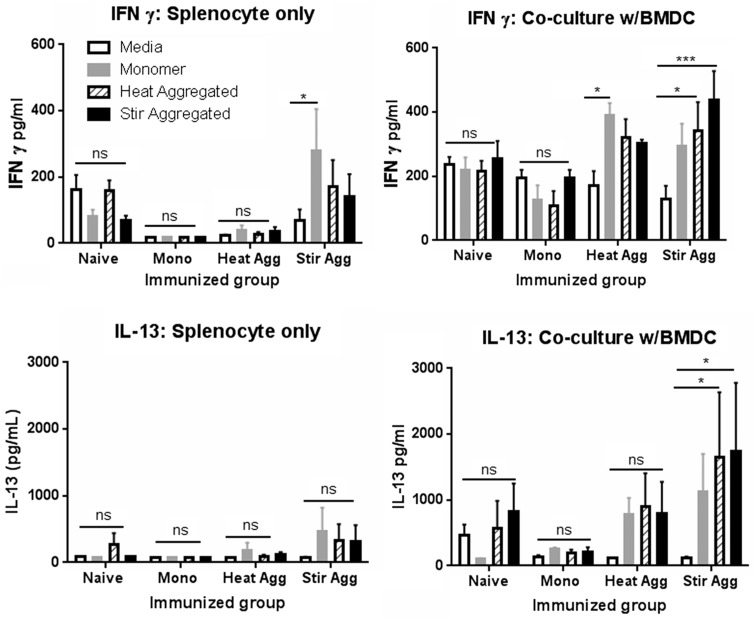
Splenocyte culture IFN-γ and IL-13 cytokine secretion following *ex vivo* scFv challenge. Splenocytes from monomer (Mono), heat (Heat Agg) or stir aggregated (Stir Agg) scFv immunized (as described in Figure 3 legend) or naïve* mice (n = 3 per group) were cultured alone (splenocyte only) and in co-culture with BMDC (w/BMDC) and challenged with 100 µg/ml monomeric scFv, heat or stir aggregated scFv or media alone. Each culture condition was performed for a single aliquot of splenocytes derived from each individual animal. Supernatants were harvested at 144 h and analyzed for the presence of IFNγ and IL-13 by cytokine specific ELISA. Data are shown as group mean ± SEM. Statistical significance of differences were calculated using a 2-way ANOVA (*P < .05, ** *P* < .01, *** *P* < .001). *Experiments with naïve mice were conducted independently but utilized the same batches of mice and protein.

To characterize further the Th1/Th2 response, culture supernatants in parallel experiments were analyzed for the presence of IFN-γ (a Th1 signature cytokine), IL-4 and IL-13 (Th2 cytokines) (Fig. 5). Secretion of IFN-γ was generally highest under splenocyte-BMDC co-culture conditions. Immunization with heat and stir aggregated scFv resulted in an increased level of IFNγ secretion from splenocyte-BMDC co-cultures in response to stimulation with scFv compared with medium alone. Monomer immunized mouse cultures did not respond to the scFv, by proliferation or by cytokine production, therefore aggregation of the immunizing material resulted in primed splenocytes that were capable of being re-stimulated by antigen in culture. Again, baseline levels of naïve cells were somewhat higher than those of immunized mice, but there was no evidence of antigen specific stimulation of these cells. Again, the nature of the scFv used for antigen challenge in culture failed to influence cytokine production levels. A similar pattern was observed for IL-13 secretion, in so far as BMDC co-culture was required for optimal production, and splenocytes derived from aggregate immunized mice displayed higher levels of cytokine production than did the monomer immunized counterparts. However, significantly higher IL-13 secretion compared with the medium control was only observed in the stir aggregate immunized co-culture group. No change in IFNγ or IL-13 production was observed with naïve splenocyte cultures. IL-4 secretion was measured in addition to IL-13 and IFN-γ; however, levels were below the limit of detection (15.6 pg/ml; data not shown).

### Aggregation of the Unrelated Protein OVA Results in Similar Th1 Skewing

**FIG. 6:**
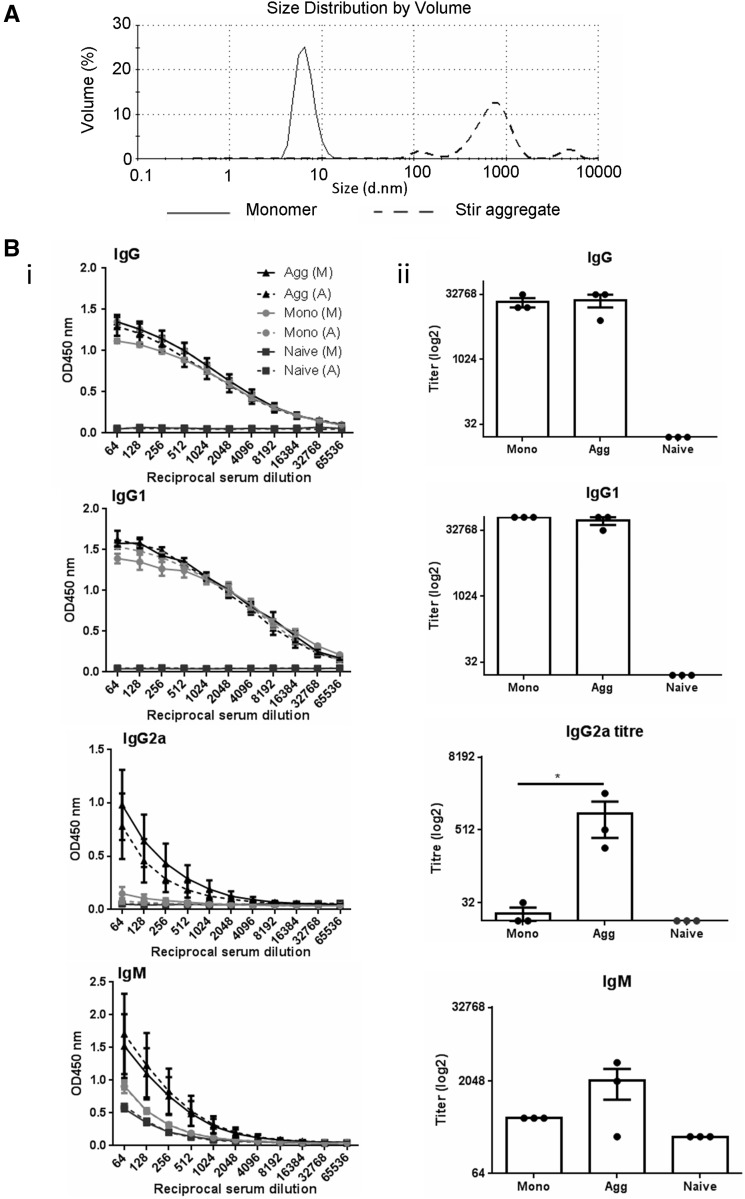
Characterization of immune responses to OVA: comparisons of monomer and stir stressed aggregates. 1 mg/ml OVA in PBS pH 7 was subjected to stir stress for 24–28 h at room temperature. **A,** The mean particle diameter was measured by DLS using the Malvern zetasizer before and after stir stress. **B,** Mice (n = 3 per group) were immunized by ip injection with 250 µl of 1 mg/ml (n = 3) monomer or stir aggregated OVA on days 0, 7, and 14 and serum isolated on day 21. Doubling dilutions of serum samples (starting dilution 1 in 64) from OVA monomer (Mono) and aggregate (Agg) immunized animals and negative control NMS samples were analyzed against OVA substrate proteins (vs monomer [M] and vs aggregated protein [A]) by ELISA for IgG, IgG1, IgG2a, and IgM anti-OVA antibody content. (i) Data are displayed as OD450 nm ±SEM for each reciprocal serum dilution (ranging from 64 to 66532).) (ii) Data are displayed with respect to antibody titer (log2) calculated as the lowest serum dilution at which 3× the ELISA substrate blank OD450 nm reading was reached. Individual titers are displayed with overall mean and SEM (vs immunizing protein only results are shown). Statistical significance of differences in antibody detection between all treatment groups against substrate were calculated using a 1-way ANOVA (*P < .05).

To determine whether aggregation and Th1 skewing was a feature solely of scFv protein or a more general property of protein aggregation *per se*, OVA was used as an alternative immunogenic protein. Stir stress was employed to aggregate OVA within the subvisible size range and DLS used for analysis. The mean diameters of OVA aggregates as a percentage of volume were: 80% approximately 0.8 µm, 10% approximately 0.1 µm, and 10% approximately 5 µm (Fig. 6A). Although these aggregates were less homogenous than observed with the scFv preparation, the mean diameter of the main population was similar to that of the scFv aggregates (1 µm). Monomer and aggregate protein preparations were administered via ip injection to mice on days 0, 7, and 14. On day 21, spleens and sera were isolated.

Analysis of the serum dilution curves and antibody titers revealed that there was no binding above the background level to either monomer or aggregate OVA substrates with naïve sera (Fig. 6B). Sera isolated from both OVA monomer or aggregate immunized mice displayed detectable IgG and IgG1 antibody, with very similar antibody titers. A significantly higher IgG2a antibody response (*P < .05) was recorded in sera from aggregate immunized compared with monomer immunized mice. There was no significant difference between IgG2a responses in naive and monomer immunized sera. Antibody titers were comparable when either aggregated or monomeric protein was used as a substrate in the analysis. In addition, anti-scFv IgM OD values and titers were higher in aggregate immunized mouse sera compared with monomer immunized mice, but not significantly different.

### Aggregation of OVA Induced Differential Cellular Responses: Proliferation and Cytokine Production

**FIG. 7:**
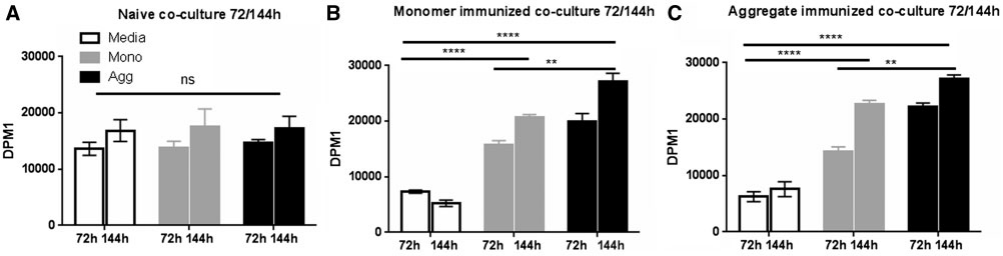
Splenocyte culture ^3^H-Thymidine incorporation following *ex vivo* OVA challenge. Splenocytes from naïve* **(A)** and monomer **(B)** or aggregate (B) immunized mice (as described in Figure 6 legend; n = 3 per group) were co-cultured with BMDC and challenged with 100 µg/ml monomeric OVA (Mono), stir aggregated OVA (Agg) or media alone. Cells were pulsed with ^3^H-thymidine 24 h before harvesting at 72 and 144 h and β scintillation counting. Each culture condition was performed in triplicate for splenocytes derived from each individual animal and a mean calculated. Proliferation measured as DPM is shown ± SEM (2 bars represent 72 and 144 h time points). Statistical significance of differences in proliferation between all groups were calculated using a 2-way ANOVA (*P < .05, ** *P* < .01, *** *P* < .001, ****p *P* < .0001). * Experiments with naïve mice were conducted independently but utilized the same batches of mice and protein.

Cellular assays with splenocyte cultures were conducted to measure antigen driven lymphocytes proliferative responses and cytokine expression. Naïve splenocyte cultures did not proliferate in response to OVA (Fig. 7A). Splenocytes from monomer and aggregate immunized mice each responded similarly in the proliferation assay (Figs. 7B and C); splenocyte-DC co-cultures proliferated significantly more in response to monomer and aggregate compared with medium controls (****P < .0001). Additionally, cultures proliferated more vigorously in response to aggregated protein than to monomer (**P < .01).

**FIG. 8:**
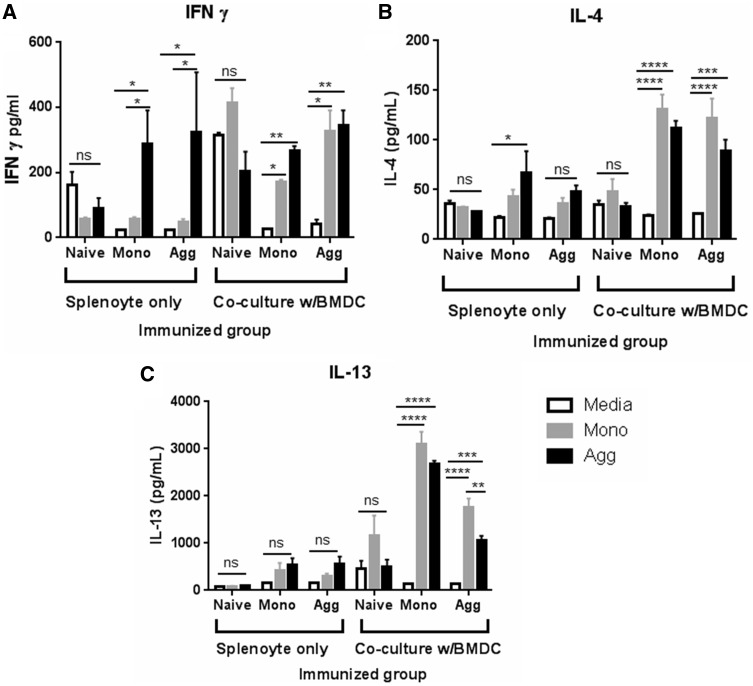
Splenocyte culture IFN-γ, IL-4, and IL-13 cytokine secretion following *ex vivo* OVA challenge. Splenocytes from naïve* and monomer (Mono) or aggregate (Agg) immunized mice (as described in Figure 6 legend; n = 3 per group) were cultured alone (splenocyte only) or in co-culture with BMDC (w/BMDC) and challenged with 100 µg/ml monomer (Mono), aggregate (Agg), or media alone. Each culture condition was performed for a single aliquot of splenocytes derived from each individual animal. Supernatants were harvested at 144 h and analyzed for the presence of **A,** IFNγ; **B,** IL-4, and **C,** IL-13 by cytokine specific ELISA. Data are shown as group mean ±SEM. Statistical significance of differences were calculated using a 2-way ANOVA (*P < .05, ** *P* < .01, *** *P* < .001, **** *P* < .0001). *Experiments with naïve mice were conducted independently but utilized the same batches of mice and protein.

Antigen-induced cytokine production by cultured splenocytes was also measured (Fig. 8). Splenocytes secreted significantly more IFN-γ when stimulated with aggregate compared with monomer or medium alone (Fig. 8A). In the presence of BMDC there was an increased level of IFNγ secretion in response to monomeric protein. IL-4 and IL-13 secretion was also increased by provision of BMDC (Figs. 8B and C): here, monomer and aggregate treated wells secreted significantly more IL-4 and IL-13 compared with medium alone controls. Stimulation with monomer challenge induced significantly higher Ievels of IL-13 production (**P < .01) compared with aggregate challenge in co-cultures from aggregate immunized mice. Naïve splenocytes did not secrete significantly more cytokine in response to OVA treatment in culture when compared with the medium alone control. These data are indicative of a Th1 skewed response against the aggregated OVA preparation, and a Th2 skewed response against the monomeric preparation.

## DISCUSSION

Although protein aggregation is widely acknowledged to have an important influence on the immunogenicity of biotherapeutics, the cellular and molecular mechanisms through which such effects are induced are unclear. Concerns about aggregate immunogenicity may be exacerbated in the future by the introduction of novel formats and/or glycoengineered molecules. For example, antigen-binding fragments (Fab), such as Lucentis (ranibizumab), and Cimzia (certolizumab pegol) have been approved (Ferrara *et al.*, 2006; Goel and Stephens, 2010), and more antibody fragments, including scFv, are in the biotherapeutics pipeline (Holliger and Hudson, 2005; Nelson, 2010). In these experiments the protein chosen to study was a humanized scFv (Edwardraja *et al.*, 2010); this was expected to provide a baseline response level of immunogenicity in mice, which could be compared with aggregate preparations. OVA was selected as a second, unrelated, antigen.

Protein aggregation can be difficult to control, and the relationship between size and immunogenicity of protein aggregates is not well understood at present. Attention is currently focused on subvisible particles, with sizes below 10 µm, due to concerns about their immunogenicity (Joubert *et al.*, 2012; Zoells *et al.*, 2012). Against this background, we aimed to generate protein aggregates that fell within the subvisible particle size range, and with a high degree of homogeneity (ie, covering a distinct size range), since aggregates often form heterogeneous populations that cover a wide size range (Das, 2012). Mild heat treatment (40 °C) or stir-stress of the scFv generated homogenous populations of aggregates in the µm range. Subvisible OVA aggregates formed by stir stress were not completely homogenous, but deemed sufficiently close in size range and homogeneity to be comparable to the scFv aggregates.

As expected, an immune response was mounted against the scFv in mice. Identical antibody binding patterns were obtained regardless of whether aggregate or monomer was used as a substrate in the ELISA, indicating that the antibody epitopes were not corrupted during heat treatment. Analysis of antibody isotype distribution revealed a Th1 skewing of the humoral immune response associated with aggregation. The data also indicated that mice immunized with aggregate had somewhat higher (but statistically insignificant) IgM titers compared with monomer. Upon antigen recognition one of the first immunoglobulins expressed by naïve B cells is IgM; further B cell activation results in class switching where the immunoglobulin constant heavy chain changes but antigen specificity remains. In these experiments relatively high total IgG and IgG2a responses demonstrated class switching of the antibody response (Kracker and Durandy, 2011); the observed levels of IgM in sera therefore were unexpected. An additional immunization was added to the protocol to determine whether IgM could be reduced with extended *in vivo* exposure. However, 3 immunizations induced strong anti-scFv IgM levels that were higher in the aggregate compared with monomer immunized mice. Higher IgM levels following aggregate administration compared with monomer immunization may suggest that the aggregate can stimulate a strong immature B cell response, in addition to B cell activation and class switching, potentially due to its structure or diversity of antigen epitopes. A lower scFv dose of 0.1 mg/ml was expected to induce a response with reduced vigor. Indeed IgG, IgG1, and IgM antibody levels in all immunized mice were reduced at the lower dose. The IgG2a response however, was still induced in mice immunized with 0.1 mg/ml aggregated scFv, and with a similar vigor of response compared with the 1 mg/ml dose. Therefore the skewing of the immune response appears more pronounced at the lower scFv dose.

Protein aggregation is known to occur under different stress conditions which can result in aggregates with different biophysical properties (Abdolvahab *et al.*, 2016). A stirring method was employed to produce aggregates mechanically, in part to imitate industrial processes where stir stresses are common. Antibody binding against the stir-aggregated substrate was similar to the monomer, indicating that antibody epitopes were not disrupted substantially with stirring. Stir and heat-stressed aggregates of the scFv were almost identical in size and antibody responses were very similar against both scFv aggregates, with a significant increase in IgG2a titers compared with monomer immunized sera. This indicates that the size range of these aggregates may be important in inducing a differential immune response or that heat and stir stresses resulted in a common misfolding of protein structure, providing aggregates with similar morphologies and therefore immunological responses. Although a similar trend was observed with both ip and sc injections, it is possible that the impact of aggregation on immunogenicity could vary according to the route of exposure, potentially due to the micro-environment or immune milieu at different sites.

Studies using cultured splenocytes from immunized mice provided further evidence of differential responses of aggregate compared with monomeric scFv. Splenocytes were also co-cultured with BMDC in an effort to improve antigen presentation and assay sensitivity for proliferation and cytokine analysis (Sun *et al.*, 2013). Indeed, we found that BMDC co-culture did improve assay sensitivity. In co-cultures from aggregate, but not monomer, immunized mice, scFv challenge resulted in increased proliferation. However, an immune response had been induced by the monomer *in vivo*, as evidenced by total IgG antibody titers which were comparable with aggregate immunized groups. This trend was also observed with results from cytokine analysis. Splenocyte cultures from aggregate immunized mice displayed enhanced secretion of both IL-13 and IFNγ with scFv stimulation; however, this effect was not observed in naïve or monomer immunized mouse cell cultures. Therefore, whilst aggregation resulted in Th1 skewing at the level of antibody production *in vivo*, an overall increase in immunogenicity was seen with respect to cytokine expression. It is worth noting that serum antibody titer represents a cumulative measure of immune activation whereas the cytokine data are a snapshot at a single time point.

OVA was chosen as an alternative candidate protein antigen to investigate; OVA differs both in size and structure to a scFv, and is a well-studied protein (Huntington and Stein, 2001), known to induce a Th2 type immune response in BALB/c mice (Carvalho Gouveia *et al.*, 2013). It was therefore of interest to determine if OVA, when aggregated in the subvisible size range, would induce a Th1 skewed immune response. Antibody analysis of immunized mouse sera did demonstrate a Th1 skewing with aggregation. Anti-OVA IgG and IgG1 levels were identical in both sets of mice; however, IgG2a antibody was significantly higher in aggregate compared with monomer immunized mouse sera. IgM binding appeared slightly higher in aggregate immunized sera, as observed with the scFv. In OVA immunized splenocyte co-cultures, aggregate culture treatment stimulated significantly higher proliferation compared with the monomer. One possible explanation for this might be that the aggregates were more easily recognized because of their size or distribution of epitopes. Furthermore, cytokine assays with splenocyte culture supernatants were consistent with Th1 skewing by aggregated OVA. Monomer and aggregate immunized splenocyte cultures secreted significantly more IFNγ in aggregate compared with monomer treated wells; however, monomer challenge induced more IFNγ secretion with the BMDC co-culture. This is likely due to improved antigen presentation in the presence of DC compared with splenocytes alone. This would also explain increased IL-4 and IL-13 secretion in co-culture compared with splenocytes alone. Increased IFNγ secretion in aggregate treated wells and IL-4 and IL-13 in monomer treated wells is in keeping with the antibody data, illustrating a Th1 skewed phenotype with aggregate treatment, and a Th2 phenotype with monomer treatment.

In the studies described here, antibody subclass distribution is a surrogate marker of divergent T cell responses. Th1 responses are typically mounted against intracellular bacteria and viruses whereas Th2 responses are associated primarily with responses in atopy and multicellular parasite infections (Berger, 2000). Viruses generally induce a Th1 type immune response in BALB/c mice (Huber and Pfaeffle, 1994). Data presented are consistent with the hypothesis that aggregates can mimic the multiple antigen copy distribution characteristics of pathogenic microbes and viruses (Kastenmueller *et al.*, 2011; Rosenberg, 2006). Vaccine studies have demonstrated Th1 skewing of immune responses by larger virus particles in BALB/c mice (Hovden *et al.*, 2005); in these experiments it was shown that a whole virus vaccine was more immunogenic and induced a more dominant Th1 antibody response compared with a split virus vaccine. In addition, BALB/c strain mice are reported to be biased towards a Th2 immune response (Fukushima *et al.*, 2006; Schulte *et al.*, 2008), so the use of these mice in this study strengthens the finding that subvisible aggregates can enhance a Th1 type immune response. It is suggested that the Th1 skewing observed with aggregation in these experiments is a direct effect of differential antigen processing and presentation.

This work shows that subvisible scFv and OVA aggregates can be achieved by heat and/or stir stress. Monomeric proteins induced a Th2 dominant immune response but when proteins were aggregated, the response gained a Th1 phenotype. This indicates that subvisible aggregates are recognized differently by immune cells, resulting in differential responses. Further work is needed to elucidate whether smaller or larger aggregates also replicate this effect, or whether other variables (ie, hydrophobicity) also play a role. Although extrapolation of our observations to humans is more difficult, our results do at least indicate that aggregation can stimulate immunological responses which differ in kind, and that the composition of aggregates could be important. The speculation is that if patients were exposed to aggregates of a similar size, the immune response may be influenced similarly, however, further work is required to understand the potential clinical implications of these findings. Although methods to predict immunodominant T cell epitopes can be useful to reflect the potential immunogenicity of a given biotherapeutic (Weber *et al.*, 2009), other considerations, such as aggregate size and biophysical properties can also influence responses. Further work will be required to define more precisely the molecular mechanisms underpinning differential immune responses to monomer and aggregate, which could then lead to an enhanced ability to predict immunogenicity.

## SUPPLEMENTARY DATA

Supplementary data are available online at http://toxsci.oxfordjournals.org/.

Supplementary Data
