# Supplementary material for: Editor’s Highlight: Subvisible Aggregates of Immunogenic Proteins Promote a Th1-Type Response
Source: Toxicol Sci. 2016 Jun 30;153(2):258–70. doi: 10.1093/toxsci/kfw121 (PMC5036615; doi:10.1093/toxsci/kfw121)
Supplement: Supplementary Data [file supp_153_2_258__index.html]

Subvisible Aggregates of Immunogenic Proteins Promote a Th1-Type Response — Editor’s Highlight: Subvisible Aggregates of Immunogenic Proteins Promote a Th1-Type Response — Supplementary Data 

# Editor’s Highlight: Subvisible Aggregates of Immunogenic Proteins Promote a Th1-Type Response

## Supplementary Data

files

- Supplementary Data - docx file
