# Supplementary material for: Editor’s Highlight: Subvisible Aggregates of Immunogenic Proteins Promote a Th1-Type Response
Source: Toxicol Sci. 2016 Jun 30;153(2):258–70. doi: 10.1093/toxsci/kfw121 (PMC5036615; doi:10.1093/toxsci/kfw121)
Supplement: Supplementary Data [file supp_kfw121_toxsci-16-0219-File010.docx]

Supplemental Data

**Title**: Subvisible aggregates of immunogenic proteins promote a Th1-type response

**Authors:** Kirsty D. Ratanji, Rebecca J. Dearman, Ian Kimber, Robin Thorpe*, Meenu Wadhwa* and Jeremy P. Derrick


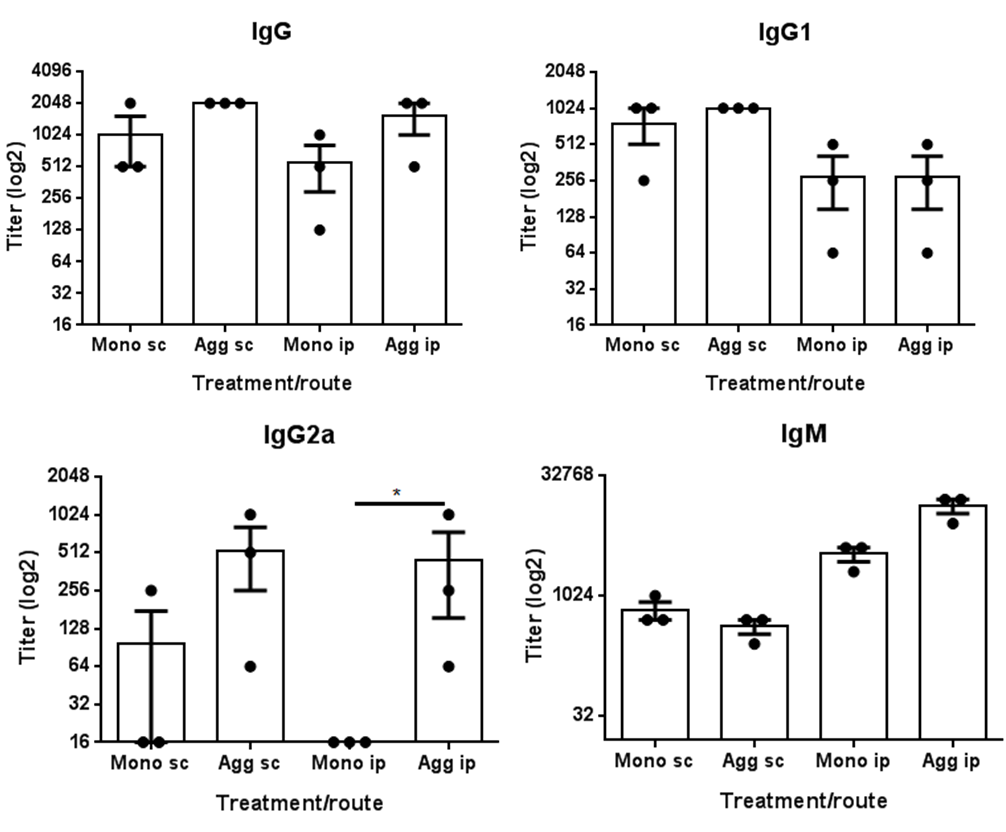


**Supplemental Figure 1. Characterisation of immune responses to scFv following ip or sc routes of administration.** Mice were immunized by ip or sc injection with monomer or heat aggregated scFv on day 0 and 7 and exsanguinated on day 14. Specific IgG subclass and IgM antibody expression in serum was assessed by ELISA. Doubling dilutions of serum samples (starting dilution 1 in 32 for IgG, 1in 128 for IgM) from scFv monomer (Mono) and aggregate (Agg) immunized animals (n=3) were analyzed against a scFv substrate (versus immunizing protein only results are shown). OD450 nm was measured; Data are displayed with respect to antibody titer (log2) calculated as the lowest serum dilution at which 3x the ELISA substrate blank OD450 nm reading was reached. Individual titers are displayed as overall mean ± SEM. Statistical significance of differences in antibody detection between all sera groups against substrate was calculated using a one way ANOVA (*p<0.05).
